# Daily consumption of Fuzhuan brick tea improves lipid profile and vascular function associated with modulation in gut microbiota composition in healthy adults: a pilot randomized controlled trial

**DOI:** 10.3389/fnut.2026.1827773

**Published:** 2026-09-28

**Authors:** Fuqiang Liu, Ying Lv, Ting Wang, Chune Li, Yudan Zhang, Lin Liu, Xin Hu, Yan Liang, Feng Zhu, Junkui Wang

**Affiliations:** 1 Department of Cardiovascular Medicine, Shaanxi Provincial People's Hospital, Xi’an, China; 2 Key Laboratory of Integrated Traditional Chinese and Western Medicine for Atherosclerosis, Research Office of Shaanxi Administration of Traditional Chinese Medicine, Xi’an, China; 3 Center for Brain Science, The First Affiliated Hospital of Xi’an Jiaotong University, Xi’an, China; 4 Center for Translational Medicine, The First Affiliated Hospital of Xi’an Jiaotong University, Xi’an, China; 5 Key Laboratory of Fu Tea Processing and Utilization, Ministry of Agriculture and Rural Affairs, Xianyang, Shaanxi, China

**Keywords:** flow-mediated dilation, Fuzhuan brick tea, gut microbiota, lipid profile, nitroglycerin-mediated dilation, vascular function

## Abstract

**Background:**

Dietary strategies play a pivotal role in the prevention of cardiometabolic diseases. Animal studies have shown that Fuzhuan brick tea (FBT), a popular post-fermented tea, enhances cardiometabolic health by modulating the gut microbiota; however, there is no definitive evidence from human trials. This 12-week randomized controlled trial investigated the effects of FBT on the lipid profile, vascular function, and gut microbiota in healthy adults.

**Methods:**

This 12-week, open-label, parallel-group, randomized controlled trial was conducted in a rural community in northern China from September to November 2021. After giving informed consent, 120 healthy adults recruited were randomly assigned to either a group consuming two daily glasses of brewed FBT infusion (totaling 12 g/day) or a group consuming an equivalent amount of water. Serum lipid levels and vascular function, as indicated by flow-mediated dilation and nitroglycerin-mediated dilation, were measured at baseline (0 weeks) and 12 weeks after intervention. Fecal samples collected at baseline, 6 weeks, and 12 weeks underwent profiling of microbiota composition by shotgun metagenomic sequencing. This trial was completed and registered at chictr.org.cn (registration number ChiCTR2100047294).

**Results:**

Compared to water, consumption of FBT resulted in significantly reduced low-density lipoprotein cholesterol levels, with improved vascular function (the co-primary outcomes). The longitudinal metagenomic analysis detected no significant alterations in richness and distribution (alpha diversity). However, a subtle but statistically detectable shift in the gut microbiota composition (beta diversity) was observed. Exploratory species-level analyses suggested increases in bacteria that produce short-chain fatty acids, such as *Roseburia hominis*, *Eubacterium saphenum*, *Eubacterium* sp. AF15 50, *Alistipes dispar*, *Blautia stercoris*, and *Lactobacillus amylolyticus*, which were correlated with the lipid profile or vascular function.

**Conclusion:**

Daily consumption of FBT demonstrated beneficial effects on lipid levels and vascular function in healthy adults, and was associated with exploratory shifts in gut microbiota composition, supporting FBT consumption is a simple, affordable, and practical dietary strategy for improving cardiometabolic health.

**Clinical trial registration:**

http://www.chictr.org.cn/, identifier ChiCTR2100047294.

## Introduction

Cardiometabolic diseases (CMDs), including diabetes, insulin resistance, obesity, stroke, nonalcoholic fatty liver disease, and cardiovascular disease, remain the primary cause of morbidity and mortality globally among all chronic non-communicable diseases (1, 2). Recognizing the urgency of addressing CMDs, global health authorities have prioritized this burden and developed policies aimed at ameliorating cardiometabolic risk factors. Clinical guidelines underscore dietary strategies as fundamental in preventing and managing CMDs (3). While many functional foods show promise in preventing and treating CMDs, the diversity in dietary habits across different countries and regions presents a considerable challenge in identifying functional foods that are compatible with local dietary practices.

Tea (*Camellia sinensis*), one of the most widely consumed beverages worldwide, offers a range of bioactive compounds that provide antioxidant, anti-inflammatory, and lipid-lowering effects (4–6). Consequently, tea has been identified as a promising functional food with potential benefits for CMDs prevention. Chinese tea can be classified into six major types based on their manufacturing processes and fermentation levels: non-fermented green tea, white tea, yellow tea, partially fermented oolong tea, fully fermented black tea, and post-fermented dark tea (7). Dark tea, including Pu-erh tea and Fuzhuan brick tea (FBT), refers to tea that undergoes microbial fermentation (8). FBT, also known as “Hu Tea” or “Jinyang Zhuan,” originated from Jingyang, Shaanxi Province, China, during the Ming Dynasty in the 16th century and is now also produced in Hunan and Guizhou provinces (9, 10). It has been a staple beverage among ethnic groups residing in the border regions of northern and western China. Notably, FBT undergoes a distinct “fungal fermentation” stage during its manufacturing process. Controlled temperature and moisture conditions facilitate the growth of yellow fungi, known as “golden flower,” within the tea. These fungi consist of a mixture of various microorganisms, with *Eurotium cristatum* being the dominant species (9, 11, 12). FBT also possesses unique functional ingredients, including polyphenols, polysaccharides, and soluble dietary fibers. In comparison to unfermented tea, such as green tea, FBT exhibits varying degrees of reduction in polyphenol content and higher levels of polysaccharides (10). In recent years, accumulating evidence in animal studies has shown that FBT and its bioactive compounds possess a diverse range of biological activities, such as antioxidant, anti-inflammatory, hypolipidemic, anti-obesity, anti-bacterial, and anti-tumor activities (10, 13, 14). Given its widespread consumption in northwest China, FBT consumption holds promise as a simple, affordable, effective, and practical approach to enhancing cardiometabolic health. However, existing evidence primarily stems from animal experiments, with limited research on humans, particularly randomized controlled trials.

With the growing recognition of tea’s diverse health benefits, researchers have begun to focus on the crucial involvement of the gut microbiota in these processes. The human gut microbiota, a complex ecosystem hosting an astonishing 100 trillion microbes across approximately 5,000 species, plays a pivotal role in host metabolism by modulating various physiological functions, including gut barrier integrity, energy uptake, and host immunity. Epidemiological and clinical studies have established connections between gut microbiota and CMDs (15–17). Mechanistic studies in animal models have further suggested a causal role of gut microbiota in the pathogenesis of CMDs through specific microbial or shared microbiota-host metabolites, identifying potential mammalian targets (15, 16). Consequently, the gut microbiome has emerged as a promising therapeutic target for CMDs. Diet significantly influences microbial abundance and functionality (18). Previous studies have demonstrated that tea consumption improves cardiometabolic health in animal models by modulating the gut microbiota (19, 20). However, studies involving humans in this area are limited and have yielded inconsistent results (21–23). Furthermore, these studies predominantly rely on the 16S rRNA gene sequencing technique. The advent of high-throughput sequencing has enabled more comprehensive analyses of the gut microbiota composition through metagenomic approaches (24, 25). Given the lack of human studies and the emergence of advanced sequencing technologies, there is a compelling need to explore the effects of tea consumption on gut microbiota using metagenomic sequencing. This approach may elucidate the relationship between changes in the gut microbiota and cardiometabolic risk factors, offering insights into potential therapeutic strategies.

In light of these considerations, we aimed to bridge the gap in human research by conducting a 12-week RCT to investigate the effects of daily FBT consumption on cardiometabolic health indicators, including lipid profile and vascular function, in healthy adults. Specifically, we employed advanced metagenomic sequencing techniques to comprehensively analyze changes in the gut microbiota composition associated with FBT consumption. Our objective was to deliver a comprehensive analysis of the changes in gut microbiota associated with FBT consumption and their correlation with cardiometabolic health indicators. This study represents a pioneering effort to explore the impacts of FBT on human health, providing valuable insights into dietary strategies for the management and prevention of CMDs.

## Methods

### Participant recruitment

In this RCT, all participants were recruited from healthy adults residing in a rural community in northern China in September 2021, ensuring uniform demographic characteristics, consistent environmental exposure, analogous dietary habits, and comparable physical activity patterns. The trial was registered at chictr.org.cn under registration number ChiCTR2100047294 on June 11, 2021. Candidates were invited for onsite screening interviews. Eligible subjects were healthy men and women between 20 and 65 years of age. Individuals were excluded from the study if they: (1) had a history of hypertension, diabetes mellitus, liver or renal diseases, enteritis, intestinal obstruction, inflammatory bowel disease, hypothyroidism, mental illness, stroke, or heart disease, or any chronic condition requiring long-term medication; (2) had a history of gastrointestinal tract surgery or disease; (3) had received prebiotics or probiotics, antibiotics, laxatives, anticholinergics, or antidiarrheal drugs more than once per week within 4 weeks before enrollment; (4) had received surgical treatment within 3 months; (5) were pregnant or lactating; (6) had participated in other clinical studies within 3 months before enrollment; (7) were regular tea drinkers or in the case of occasional tea drinkers, had not adhered to a 2-week washout period prior to starting the intervention; or (8) had other health issues unsuitable for participation. A total of 342 candidates were screened, and 120 of them were eligible and consented to participate in the study. The institutional ethics committee of Shaanxi Provincial People’s Hospital approved the study protocol, and each participant gave written informed consent. All procedures were performed in accordance with institutional guidelines.

### Intervention strategies

The present study was an open-label, parallel-group RCT. Participants were randomly allocated to the FBT group (*n* = 60) or the control group (*n* = 60) in a 1:1 ratio. Randomization was conducted using computer-generated random numbers, employing a stratified block design stratified by sex (male/female), with a block size of 6 within each stratum. Allocation was concealed using sequentially numbered, opaque, sealed envelopes (SNOSE). The randomization sequence was generated by Yudan Zhang, who was not involved in participant enrollment or intervention assignment. Participants in the FBT group consumed 2 × 300 mL of FBT infusion per day (2 standard cups of 300-mL hot water, each containing a 6-g FBT capsule) for 12 weeks. The control group consumed an equivalent volume of hot water for the same intervention period. All participants were instructed to adhere to their regular diet and activity patterns. They were also required to complete a three-day dietary record and the International Physical Activity Questionnaire at baseline (Week 0), Week 6, and Week 12. Participant compliance was monitored through WeChat check-ins and monthly follow-up visits. Medication and supplement usage, as well as adverse events, were self-reported throughout the study.

### Preparation of FBT

FBT was purchased from Xianyang Jingwei Fu Tea Co. Ltd., China, and underwent a standardized grinding process to prepare a homogenous fine powder. Then, the powder was portioned into precise 6-g single-serve tea capsules (sealed containers, not intended for oral ingestion). A dedicated brewing device was designed and calibrated to deliver a fully standardized preparation protocol: each capsule was placed into the device, which automatically dispensed a fixed volume of 300 mL water at a constant temperature of 90 °C and maintained a precise brewing duration of 5 min. This dedicated apparatus ensured uniform extraction conditions across all participants and all administration occasions throughout the 12-week intervention, thereby minimizing inter-occasion variability in bioactive compound delivery. Participants consumed only the brewed tea infusion; the spent capsule and residual tea powder were discarded. FBT contains 23.29 ± 0.09 mg/g of free amino acids (FAAs), 52.19 ± 0.51 mg/g of soluble sugars (SSs), 3.62 ± 0.03% and 32.55 ± 0.7 mg/g of caffeine (CAF), 103.18 ± 0.9 mg/g of tea polyphenols (TPs), and 16.92 ± 0.79 mg/g of total flavonoids (TFs). Additionally, it contains (16.5 ± 0.71) × 10^5^ CFU/g of *Eurotium cristatum*. The FFAs, CAF, and TPs were determined according to the spectrophotometry method used in our previous study. The SSs and TFs were measured using colorimetric methods based on anthrone and aluminum chloride. The TPs and TFs were quantified based on the equivalent content of gallic acid and rutin, respectively (26). The 12 g/day dose was selected based on traditional FBT consumption patterns in northwestern China (15–30 g/day) (9, 10), human equivalent doses derived from animal studies (approximately 8–15 g/day).

### Specimen collection

Stool samples were collected at baseline, Week 6, and Week 12. These samples were initially stored in the participants’ home freezers at −20 °C and wrapped in ice packs before being transported on ice to the research laboratory for storage at −80 °C. Fasting blood samples were collected from an antecubital vein by a phlebotomist using standard clinical procedures at baseline and Week 12. For serum analysis, blood was collected in serum-separating tiger top tubes, centrifuged at 1,200 × g for 10 min, aliquoted, and stored at −80 °C. For plasma analysis, blood was drawn into ethylenediaminetetraacetic acid tubes, centrifuged under the same conditions, aliquoted, and similarly stored at −80 °C.

### Biochemical and hematologic parameter analysis

Fresh serum samples were promptly dispatched to Shaanxi Provincial People’s Hospital on the same day of collection for the meticulous analysis of lipid-lipoprotein profiles using well-established standard techniques. The total cholesterol (TC) levels were determined by an enzymatic colorimetric method using cholesterol esterase, cholesterol oxidase, peroxidase, and chromagen 4-aminophenazone/phenol. Triglyceride (TG) levels were determined by an enzymatic colorimetric method using lipoprotein lipase glycerokinase, glycerophosphate oxidase, and chromagen 4-aminophenazone /N-ethyl-N -(3-sulphopropyl)-m-amisidine. The high-density lipoprotein cholesterol (HDL-C) levels were determined by an immunoinhibition assay. The low-density lipoprotein cholesterol (LDL-C) levels were directly measured in fasting serum using a homogeneous direct enzymatic method (hydrogen peroxide scavenging method) on a Hitachi LABOSPECT 008AS automated clinical chemistry analyzer (Hitachi High-Technologies Corporation, Tokyo, Japan), using reagents from Maccura Biotechnology (Chengdu, China), according to the manufacturer’s instructions. The investigators analyzing the samples were blinded to the identities of the samples.

### Vascular function measurements

To evaluate the vascular function of participants, flow-mediated dilation (FMD) was conducted in line with established guidelines. Prior to the FMD test, participants were instructed to abstain from smoking, consuming food, or ingesting caffeine-containing beverages for 12 h. The FMD test was scheduled between 9 a.m. and 10 a.m. Participants were required to lie down and rest for a minimum of 15 min in a calm environment with a controlled temperature of 25 °C. Using a high-resolution ultrasound device (Vivid I; GE Healthcare, Trondheim, Norway), the diameter of the brachial artery was measured over a 5 cm segment. Baseline measurement was taken for 30 s, followed by measurement during peak reactive hyperemia, which occurred after the release of a blood pressure cuff that had been placed around the forearm and inflated to 50 mmHg above systolic blood pressure for 5 min. The results were quantified as the percentage increase in arterial diameter from baseline to the maximum post-cuff release. To assess endothelium-independent vasodilation, the nitroglycerin-mediated dilation (NMD) was measured. After a rest period of at least 15 min following the FMD test, the arterial diameter response was recorded following the sublingual administration of 0.5-mg nitroglycerin. The NMD was calculated based on the increase in arterial diameter measured 5 min post-nitroglycerin administration, expressed as a percentage of the baseline diameter. The investigators conducting the vascular function measurements were blinded to the group allocation.

### DNA preparation and shotgun metagenomics sequencing

Metagenomic sequencing and bacterial DNA extraction were conducted using established protocols. Bacterial DNA from fecal samples was extracted using the MagBeads FastDNA™ Kit (MP Biomedicals, Shanghai, China), following the manufacturer’s instructions. The DNA concentrations were determined using NanoDrop 2000, and their integrity was assessed by 1% agarose gel electrophoresis using the KAPA HyperPlus PCR-free reagent (Illumina, CA, USA). The DNA was then sheared to an average size of approximately 400 bp for paired-end library construction. Qubit 2.0 (Thermo Fisher, Massachusetts, USA) was used to quantify the DNA samples, and Agilent 2,100 (Agilent, Palo Alto, USA) was used to verify the read quality. Libraries from all samples were pooled, ensuring a total DNA amount of 300 ng in a volume of ≤48 μL. Following heat denaturation, single-strand circularization, enzymatic digestion, and purification, shotgun metagenomic sequencing was executed on the BGI platform (MGESEQ-T7, Shenzhen, China) by Shaan Probiomicros Co., Ltd. (Xi’an, China). Raw reads underwent quality control using fastp (v0.23.0) with parameters set to length_required = 50 and n_base_limit = 2. Host reads were subsequently filtered out using Bowtie2 software (v2.3.5.1) based on human genomes.

### Metagenomic data analysis

Following rigorous quality control of the sequencing data, MetaPhlAn4 software was used to annotate the metagenomic data and analyze the composition of microbial communities to obtain relative abundance information at different taxonomic levels (27). Microbiota richness and distribution were assessed using α-diversity indices, using three different indices (i.e., Shannon, inverse Simpson, and Simpson). The statistical significance of alpha diversity between the two groups at different time points was evaluated by the Mann–Whitney U test or Student’s *t*-test. Compositional disparities between the two groups were discerned through principal coordinates analysis (PCoA) based on the Bray–Curtis distance. PERMANOVA was performed with group as the main factor and age, gender, alcohol intake, and smoking as covariates to assess the independent effect of FBT on beta diversity. To verify that PERMANOVA results were not confounded by differences in within-group dispersion, the betadisper test was performed on the Bray-Curtis distance matrix at week 12. The influence of group categorization and other variables on the gut microbiota was evaluated using permutational multivariate analysis of variance. Longitudinal analysis was performed using SplinectomeR (28). Differences in taxon abundances over the course of the study were determined using the permuspliner function of SplinectomeR, a permutation-based package in R utilizing weighted local polynomials to assess group differences in longitudinal microbiota data, with multiple time points applied per subject.

For functional profiling, HUMAnN3 was utilized to obtain information on species abundance, metabolic pathways, and functional modules of microorganisms. The Gut Metabolic Modules (GMMs) database, including 103 metabolic pathways, was employed to analyze and annotate microbial functional changes. The GMMs database captures bacterial and archaeal metabolic pathways specific to the human intestinal environment, emphasizing anaerobic fermentation processes (29). The GMM abundance was estimated using Bowtie2 (v2.3.5.1) (30), which was constructed using data from an extensive literature review and metabolic databases, including Kyoto Encyclopedia of Genes and Genomes (KEGG) and MetaCyc. A species was deemed to possess a GMM if it harbored at least two-thirds of the necessary orthologs according to the KEGG of a module. To determine the abundance of each module, we calculated the median abundance of these KEGG orthologs within the pathway that exhibited the maximum coverage.

### Statistical analysis

This study was designed as an exploratory pilot study. The sample size of 120 participants was determined based on feasibility and available resources rather than a formal sample-size calculation. The primary outcome of this study was the change in lipid–lipoprotein profiles and vascular function parameters. Secondary outcomes were changes in the gut microbiota composition after FBT intervention. All primary and secondary efficacy analyses were performed on the per-protocol population. All data were checked for normality using the Shapiro–Wilk test before further analyses. Continuous outcomes were analyzed using the *t*-test or Wilcoxon rank sum test, as appropriate. Dichotomous outcomes were analyzed using Fisher’s exact test. The comparison of lipid–lipoprotein profiles and vascular function parameters in the stage before and after the intervention in each group was performed using a paired sample *t*-test. To account for the repeated-measures design and within-subject correlations, linear mixed-effects models (LMM) were employed to evaluate the effects of FBT on the primary outcomes. For each outcome, group (FBT vs. control), time (baseline vs. week 12), and their interaction were included as fixed effects, with age, sex, alcohol intake, and smoking as covariates, and subject as a random intercept. The group × time interaction was used to assess whether FBT produced significantly different temporal changes compared with the control group. As a sensitivity analysis, analysis of covariance (ANCOVA) was additionally performed for primary outcomes, with the post-intervention value as the dependent variable, group assignment as the fixed factor, and the corresponding baseline value as the covariate. A second model further adjusted for age, sex, and BMI. Longitudinal analysis was performed using SplinectomeR. A *p*-value of 0.05 was deemed statistically significant. The species taxonomic level was selected for analysis. The connection between species and clinical data was assessed using Spearman’s rank correlation. Correlation analyses were performed using pooled data from baseline and week 12 to capture both pre- and post-intervention variability. All correlation *p*-values were adjusted for multiple testing using the Benjamini-Hochberg FDR procedure. To address the potential non-independence of pooled repeated observations, a sensitivity analysis was additionally performed using week-12 data only. Statistical analysis and visualization were carried out in the R environment V.3.4.2^1^.

## Results

### Demographic and clinical characteristics

The CONSORT diagram and flowchart of participants through the study are depicted in Figure 1. A total of 342 individuals were initially screened for eligibility, of whom 120 were eventually enrolled. During the 12-week intervention, 10 participants discontinued (FBT group, *n* = 5; control group, *n* = 5). The reasons for discontinuation were hospitalization due to infection (*n* = 1), gastrointestinal discomfort unrelated to the intervention (*n* = 2), initiation of statin therapy (*n* = 3), initiation of antibiotic therapy (*n* = 3), and loss to follow-up (*n* = 1). All statistical analyses were performed on the per-protocol population, comprising the 110 participants who completed the intervention and provided valid biological samples at both baseline and week 12. The baseline characteristics, including anthropometric and blood biochemical traits, were well-balanced between the two groups (Table 1). Baseline characteristics did not differ significantly between completers and non-completers (Supplementary Table 1). With respect to potential confounders, neither three-day dietary records nor IPAQ-assessed physical activity showed significant between-group differences at any time point, nor significant within-group changes from baseline to week 12 (all *p* > 0.05; Supplementary Table 2), indicating that dietary habits and physical activity levels remained stable throughout the intervention in both groups.

**Figure 1:**
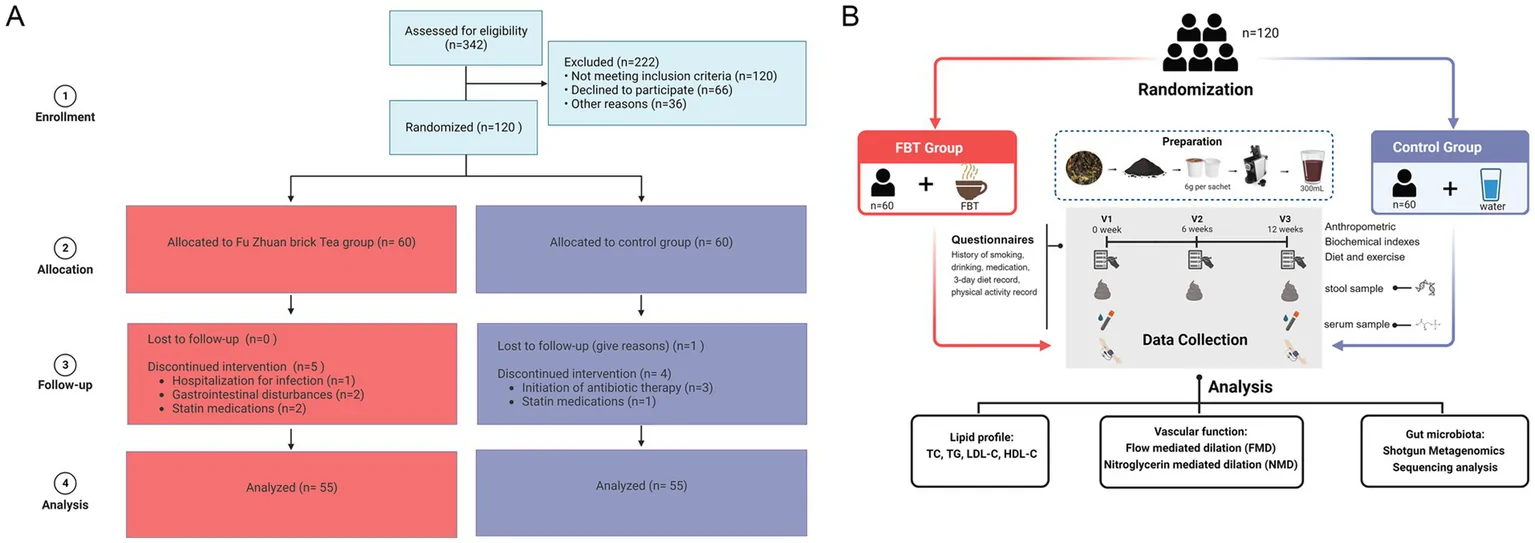
The CONSORT study flow diagram and study design. **(A)** Participant flowchart. **(B)** Study design.

**Table 1:** Demographic information.

Variable	Experimental group (*n* = 55)	Control (*n* = 55)	Statistic Value	*p* value
Age (years), Mean ± SD	53.82 ± 8.40	53.98 ± 7.98	0.108	0.917^a^
Sex, *n* (male/ female)	31/24	32/23	0.037	0.874^b^
Alcohol use, *n* (y/*n*)	25/30	22/33	0.334	0.563^b^
Smokers, *n* (y/*n*)	16/39	19/36	0.377	0.539^b^
BMI, kg/m2	25.52 ± 2.95	24.45 ± 3.08	1.857	0.066^a^
SBP, mmHg	122.41 ± 13.31	126.02 ± 13.42	1.412	0.161^a^
DBP, mmHg	77.71 ± 8.16	79.54 ± 6.56	1.3	0.196^a^
HbA1c, %	5.83 ± 0.31	5.79 ± 0.28	0.672	0.503^a^
BUN, mmol/L	5.36 ± 1.44	5.38 ± 1.25	0.129	0.898^a^
SCr, μmol/L	58.43 ± 12.84	57.41 ± 11.48	0.438	0.663^a^
AST, U/L	20.85 ± 7.67	21.04 ± 6.74	0.132	0.895^a^
ALT, U/L	19.45 ± 10.93	18.88 ± 10.45	0.285	0.776^a^
Total cholesterol, mmol/L	4.82 ± 1.06	5.06 ± 0.85	1.315	0.191^a^
Triglycerides, mmol/L	1.99 ± 1.79	1.58 ± 0.65	1.558	0.122^a^
LDL cholesterol, mmol/L	3.02 ± 0.83	3.17 ± 0.77	0.983	0.328^a^
HDL cholesterol, mmol/L	1.08 ± 0.29	1.18 ± 0.28	1.97	0.051^a^

*n*, number of participants; BMI, body mass index; SBP-DBP, systolic and diastolic blood pressure; LDL-HDL, low and high density lipoprotein; AST-ALT, aspartate and alanine transaminase; SCr, serum creatinine.

a Statistical analysis by *t*-test.

b Statistical analysis by chi square test.

### Effects of FBT on serum lipids

At baseline, serum lipid levels, including TC, TG, LDL-C, and HDL-C, were comparable between the FBT and control groups. Within the FBT group, LDL-C significantly decreased after the 12-week intervention (mean Δ = −0.36 mmol/L, 95% CI: −0.47 to −0.26, *p* < 0.001), whereas no significant change was observed in the control group (mean Δ = +0.03 mmol/L, *p* = 0.705) (Figure 2A). HDL-C increased significantly in both the FBT group (mean Δ = +0.14 mmol/L, *p* < 0.001) and the control group (mean Δ = +0.08 mmol/L, *p* = 0.004). TG showed a decreasing trend in the FBT group (mean Δ = −0.43 mmol/L, *p* = 0.083) but no significant change in the control group (mean Δ = −0.05 mmol/L, *p* = 0.544). TC remained stable in both groups (FBT: mean Δ = +0.08 mmol/L, *p* = 0.270; control: mean Δ = −0.03 mmol/L, *p* = 0.779). To assess between-group differences, linear mixed-effects models were employed. The group × time interaction was significant for LDL-C (χ^2^ = 16.73, *p* < 0.001), indicating that FBT consumption produced a significantly greater reduction over time compared with the control group (Δ = −0.40 mmol/L), independent of age, sex, alcohol intake, and smoking. For TG, the group × time interaction was not significant (χ^2^ = 2.13, *p* = 0.145; Δ = −0.38 mmol/L); notably, age (*p* = 0.021) and sex (*p* = 0.032) were significant covariates, suggesting that the within-group decreasing trend in TG may be partially confounded by these factors. The group × time interactions were also not significant for TC (χ^2^ = 0.82, *p* = 0.364; Δ = +0.10 mmol/L) or HDL-C (χ^2^ = 3.77, *p* = 0.052; Δ = +0.06 mmol/L). None of the covariates were significant predictors for LDL-C (Supplementary Table 3).

**Figure 2:**
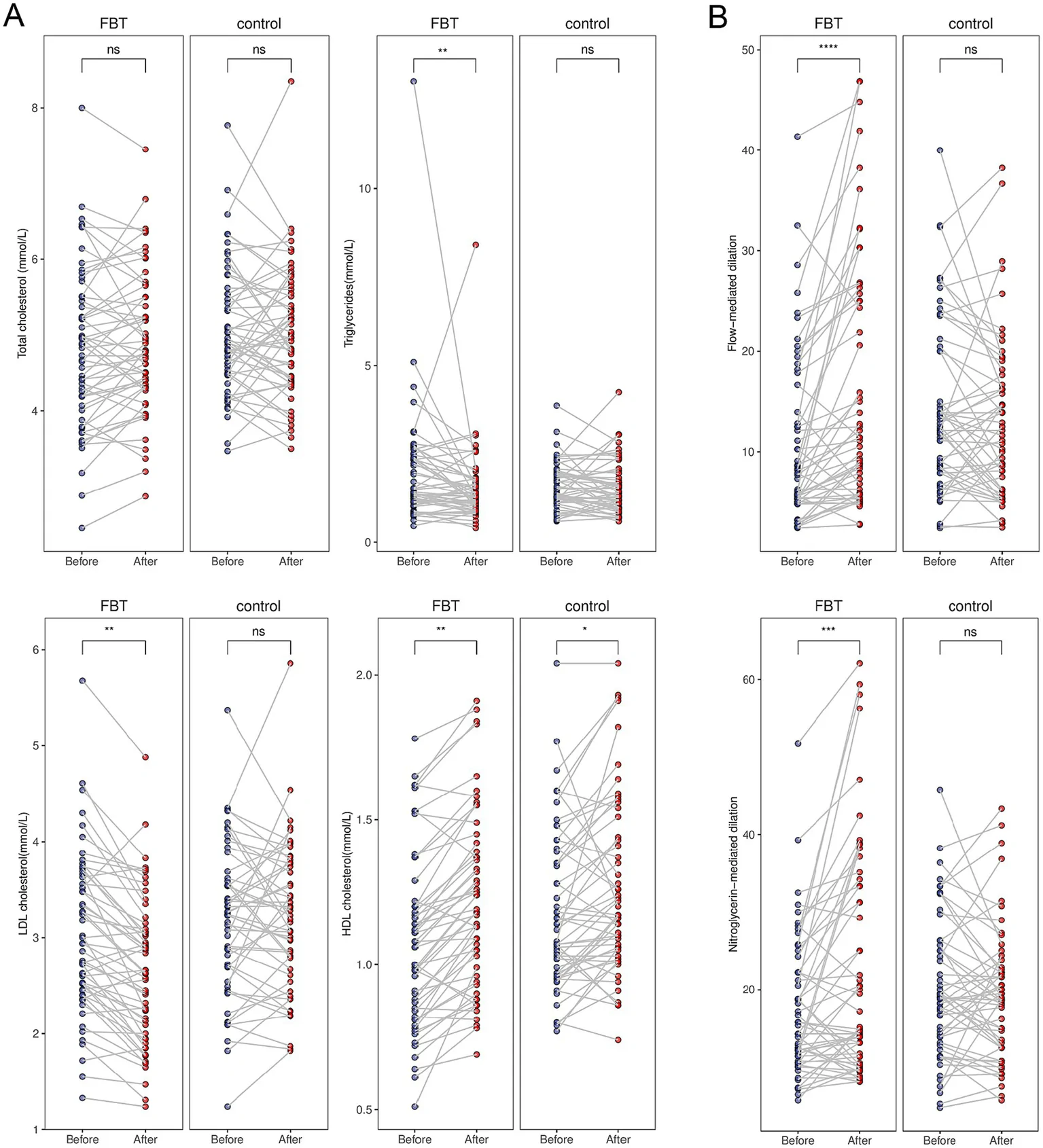
Effects of Fuzhuan brick tea on serum lipids **(A)** and vascular function **(B)** in healthy adults. **(A)** Serum levels of total cholesterol, triglycerides, low-density lipoprotein cholesterol, and high-density lipoprotein cholesterol before and after the 12-week intervention in the FBT and control groups. **(B)** Flow-mediated dilation and nitroglycerin-mediated dilation before and after the 12-week intervention in the FBT and control groups. Data are presented as individual values with lines connecting paired samples. Within-group comparisons (before vs. after intervention) were performed using paired *t*-tests. ns, not significant; *p < 0.05; **p < 0.01; ***p < 0.001; ****p < 0.0001 vs. baseline within each group.

### Effects of FBT on vascular function

FMD and NMD were measured to evaluate the effects of FBT on vascular function. At baseline, FMD values were lower in the FBT group than in the control group (10.97 ± 5.53 vs. 14.39 ± 8.52%). Within the FBT group, FMD significantly increased after the 12-week intervention (mean Δ = +5.45%, 95% CI: 3.14–7.76, *p* < 0.001), whereas no significant change was observed in the control group (mean Δ = −1.21%, 95% CI: −2.89 to 0.46, *p* = 0.153) (Figure 2B). Similarly, NMD significantly increased in the FBT group (mean Δ = +5.35%, 95% CI: 2.60–8.10, *p* < 0.001) but not in the control group (mean Δ = −0.71%, 95% CI: −2.51 to 1.09, *p* = 0.434). To assess between-group differences, linear mixed-effects models were employed. The group × time interaction was significant for both FMD (χ^2^ = 21.89, *p* < 0.001; Δ = +6.66%) and NMD (χ^2^ = 13.67, *p* < 0.001; Δ = +6.06%), indicating that FBT consumption produced significantly greater improvements in vascular function over time compared with the control group, independent of age, sex, alcohol intake, and smoking. None of the covariates were significant predictors for either FMD or NMD (Supplementary Table 3). To address the baseline difference in FMD between groups, we performed ANCOVA with week-12 FMD as the dependent variable and baseline FMD as the covariate. After adjusting for baseline FMD, the between-group difference remained significant (adjusted mean difference: 6.27%; 95% CI: 3.40, 9.14; *p* < 0.001; Cohen’s d = 0.59). Further adjustment for age, sex, and BMI yielded consistent results (adjusted mean difference: 6.45%; 95% CI: 3.44, 9.45; *p* < 0.001; Supplementary Table 4). Similarly, ANCOVA for NMD showed a significant between-group difference (adjusted mean difference: 5.92%; 95% CI: 2.63, 9.20; *p* < 0.001). These findings confirm that the observed vascular function improvements are not attributable to baseline imbalance.

### Effects of FBT on gut microbiota richness, diversity, and composition

Shotgun metagenomics sequencing was conducted to determine the impact of FBT on gut microbiota composition. On average, 14.81 ± 4.09 Gb of raw reads were obtained per sample. Across all samples, we identified 16 phyla, 34 classes, 60 orders, 118 families, 373 genera, and 1,019 species. Alpha diversity, measured using Shannon, inverse Simpson, and Simpson indices, showed no significant differences between groups at any taxonomic level throughout the study (Figure 3A). This finding was confirmed by longitudinal analysis using SplinectomeR, indicating that FBT did not affect alpha diversity (Figure 3A).

**Figure 3:**
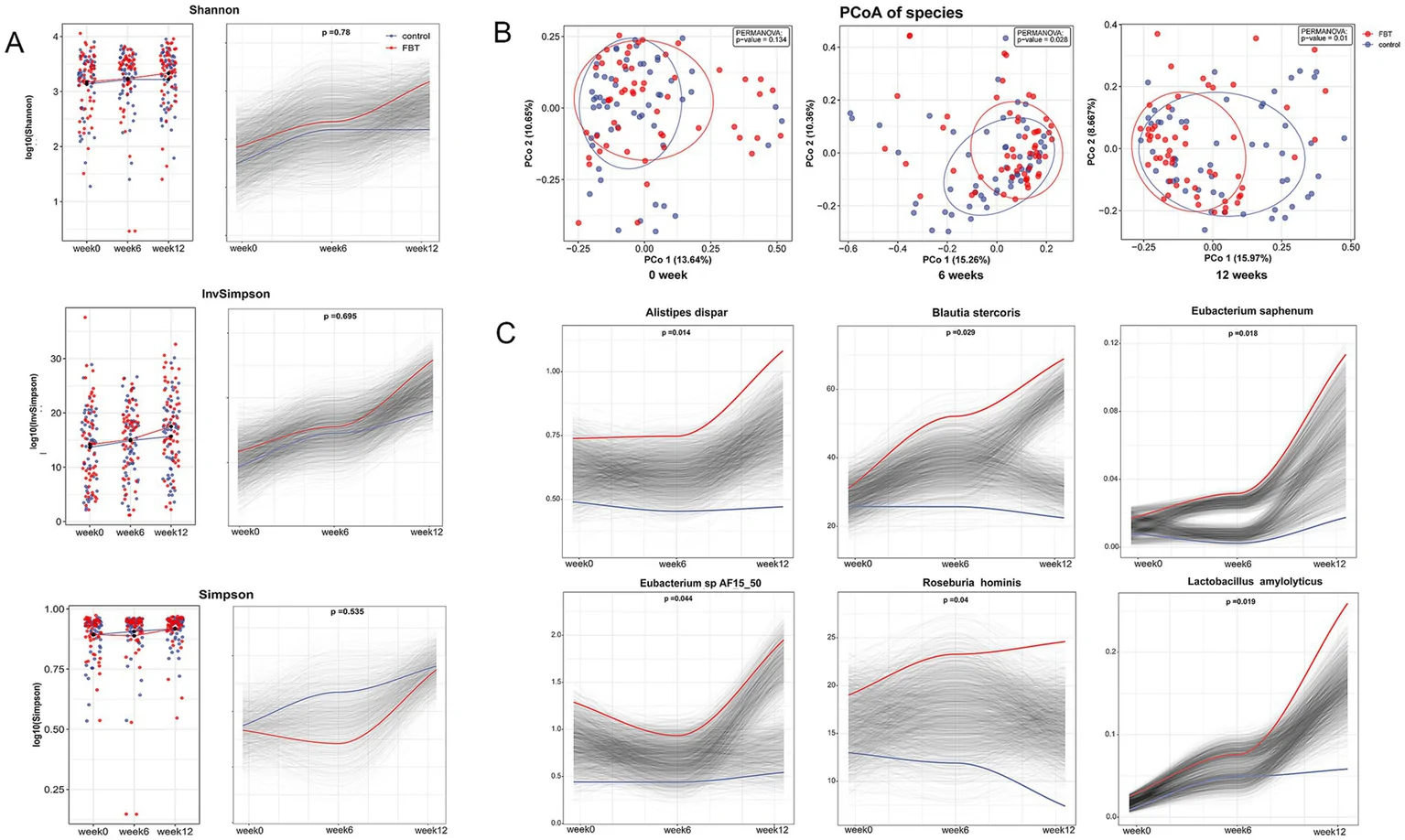
Impact of Fuzhuan brick tea on the gut microbiome over time. **(A)** Longitudinal trajectories of Shannon index, InvSimpson index, and Simpson index, indicating alpha (within-sample) diversity over time; *p* values are from trend comparison using SplinectomeR (999 permutations). **(B)** PCoA depicting beta (between-sample) diversity based on Bray–Curtis dissimilarity at each time point, with confidence ellipses for each group; *p* values are from PERMANOVA (99,999 permutations) adjusted for age, gender, alcohol intake, and smoking. **(C)** Longitudinal trajectories of the relative abundances of 6 SCFA-producing bacteria over time; *p* values are from trend comparison using SplinectomeR (999 permutations). The lines represent the group splines for the FBT group (red) (*n* = 55) and the control group (blue) (*n* = 55). Species-level longitudinal differences were assessed using permuspliner (unadjusted *p* < 0.05); these findings are exploratory and did not survive FDR correction across all tested species (see Supplementary Table 5).

Principal coordinates analysis (PCoA) of gut species profiles revealed subtle but statistically detectable shifts in bacterial community composition over the intervention period (Figure 3B). At baseline, no significant differences in beta diversity (community composition) were observed between groups after adjustment for age, gender, alcohol intake, and smoking (R^2^ = 0.011, *p* = 0.134). Small but significant group differences emerged at Week 6 (R^2^ = 0.016, *p* = 0.028) and Week 12 (R^2^ = 0.019, *p* = 0.01), indicating that FBT consumption was associated with modest alterations in overall community composition. The betadisper test confirmed no significant difference in multivariate dispersion between groups at week 12 (*F* = 0.9931, *p* = 0.321), supporting the reliability of the PERMANOVA findings. The predominant phyla included *Firmicutes, Bacteroidetes, Actinobacteria, and Proteobacteria*. Although baseline composition was similar between groups, the relative abundance of *Actinobacteria* was lower, and *Verrucomicrobia* was higher in the FBT group than in the control group (Supplementary Figure 1).

We also examined longitudinal species-level differences and identified 77 bacterial species with nominally significant differences between groups (unadjusted *p* < 0.05; Supplementary Figure 2). After Benjamini-Hochberg FDR correction across all tested species, none remained statistically significant (Supplementary Table 5). Therefore, all species-level findings reported herein should be interpreted as exploratory. Among these exploratory findings, the FBT group exhibited a discernible upward trend in the relative abundance of several short-chain fatty acid (SCFA)-producing bacteria, including *Roseburia hominis*, *Eubacterium saphenum*, *Eubacterium* sp. AF15 50, *Alistipes dispar*, and *Blautia stercoris* (Figure 3C). Additionally, *Lactobacillus amylolyticus* was more abundant in the FBT group, showing a suggestive longitudinal difference compared to the control group (Figure 3C).

### Effects of FBT on the functional characterization of gut microbiota

We also investigated potential functional changes in gut microbiota. To estimate the abundance of metabolic pathways, we mapped our metagenomic reads to functional orthologues from the KEGG and GMM databases. Using Bray–Curtis dissimilarity-based PCoA, we analyzed variations in 103 GMM metabolic pathways across different groups and time points. This analysis revealed a subtle but statistically detectable difference in pathway distribution between the two groups at Week 12 (Figure 4A).

**Figure 4:**
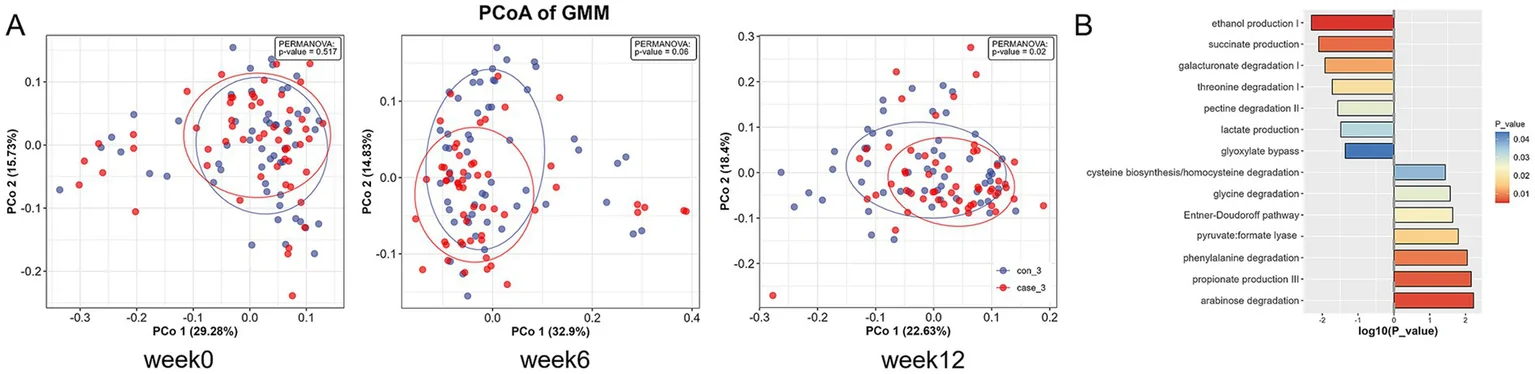
Impact of Fuzhuan brick tea on the functional characterization of the gut microbiome in healthy adults over time. **(A)** PCoA depicting the differences in functional characterization based on Bray–Curtis dissimilarity at each time point; *P* values are from PERMANOVA (99,999 permutations) adjusted for age, gender, alcohol intake, and smoking. **(B)** Bar chart depicting metabolic pathways showing nominal differential abundance (permuspliner *p* < 0.05) between the FBT and control groups over the 12-week intervention; Pathways are ordered by permuspliner *p*-value.

We further employed SplinectomeR for a longitudinal analysis of GMM pathway abundance between the FBT and control groups. Fourteen metabolic pathways showed nominal differences in abundance (permuspliner *p* < 0.05) (Figure 4B). Specifically, the FBT group showed enrichment in seven pathways: cysteine biosynthesis/homocysteine degradation, glycine degradation, the Entner-Doudoroff pathway, pyruvate lyase, phenylalanine degradation, propionate production III, and threonine degradation I. Conversely, the control group showed higher abundances in another set of seven pathways, including pectin degradation II, lactate production, glyoxylate bypass, succinate production, galacturonate degradation I, and ethanol production I. However, none remained significant after Benjamini–Hochberg false discovery rate (FDR) correction (Supplementary Table 6). These pathway-level findings should therefore be interpreted as exploratory.

### The gut metagenome is associated with lipid profile and vascular function

Lastly, we explored the correlations between gut microbial taxa, functions, and clinical parameters, including lipid profile and vascular function. We performed a three-pronged correlation analysis between microbial species, functions, and clinical parameters using Spearman’s rank correlation (Figure 5). Among the 77 differentially abundant bacterial species (exploratory, unadjusted *p* < 0.05), 22 species showed significant correlations with lipid profile parameters, while 10 species were significantly associated with vascular function. Five SCFA-producing bacteria, including *Roseburia hominis*, *Eubacterium saphenum*, *Eubacterium* sp. AF15 50, *Alistipes dispar*, and *Blautia stercoris,* exhibited significant negative correlations with LDL-C levels, which were enriched in the FBT group. *Eubacterium saphenum* and *Lactobacillus amylolyticus* were not only negatively correlated with LDL-C levels but also positively correlated with FMD and NMD. Furthermore, the propionate production III pathway (one of the nominally differentially abundant GMMs) showed a negative correlation with LDL-C levels and a positive correlation with FMD and NMD. A sensitivity analysis restricted to week-12 data alone produced a correlation pattern highly consistent with the primary analysis (Supplementary Figure 3).

**Figure 5:**
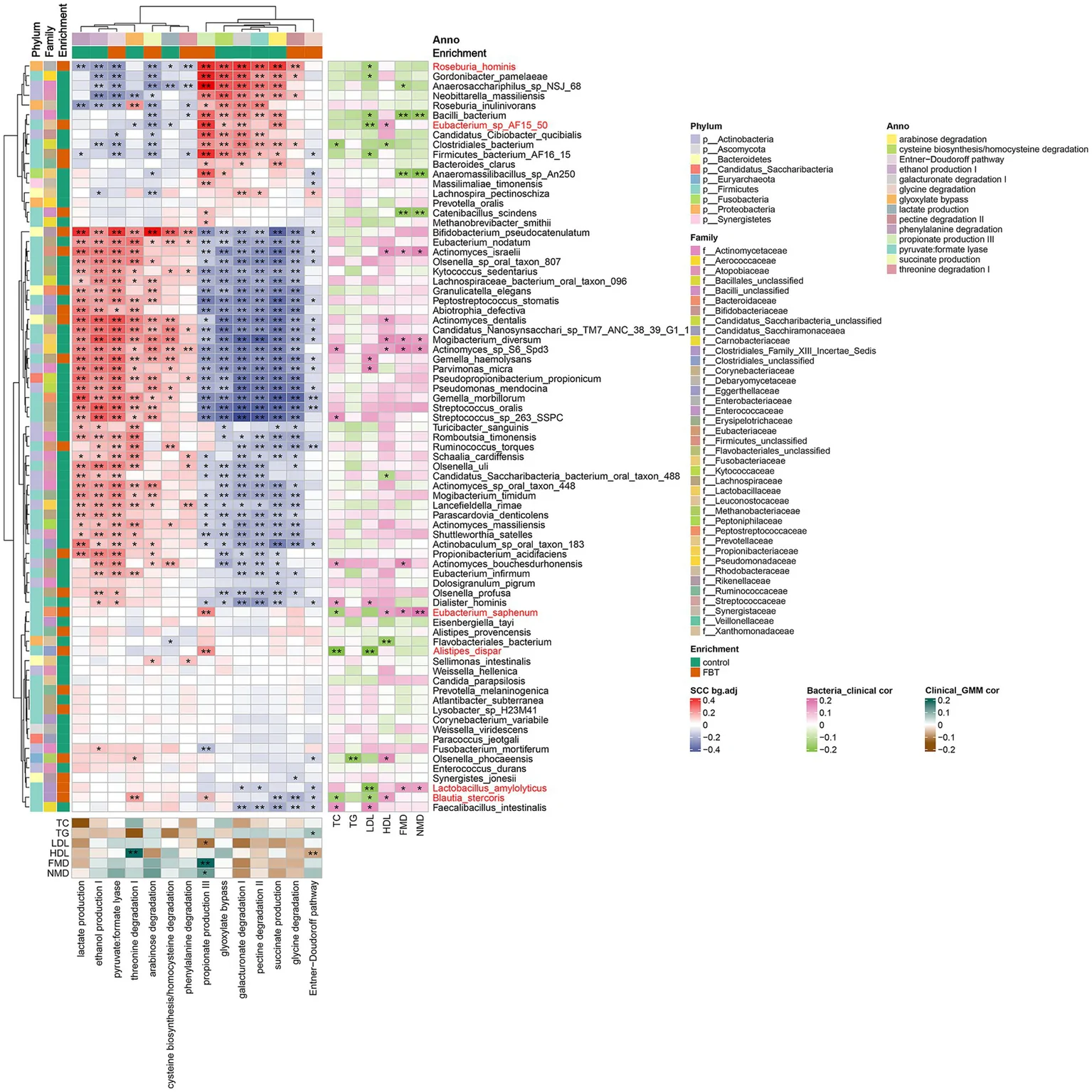
Three-pronged association heatmap of bacterial species, GMM pathways, and clinical parameters. The left panel denotes the family-level and phylum-level taxonomy and directionality of enrichment in the FBT group (red) and control group (green) for species. The centered heatmap denotes the Spearman correlation between each species and each GMM pathway (red: positive correlation; blue: negative correlation). The right panel denotes the Spearman correlations between bacterial species and clinical parameters, colored by positive (pink), negative (green), or nonsignificant correlation (white). The bottom panel denotes the significant association between the GMM pathways and clinical parameters (cyan: positive correlation; brown: negative correlation). ‘*’ denotes FDR *p* < 0.05; ‘**’ denotes FDR *p* < 0.01.

## Discussion

In the current study, we observed that daily FBT consumption for 12 weeks exhibited significant potential in modulating serum lipid levels, notably reducing LDL-C levels. Furthermore, FBT consumption enhanced both endothelium-dependent and endothelium-independent vascular dilation functions, indicating a beneficial impact on vascular health. Additionally, our results revealed that FBT consumption led to favorable shifts in the gut microbiota composition, with nominally enriched SCFA-producing taxa and related functional modules; although these taxonomic and pathway-level differences did not survive FDR correction, the directionality of the findings is consistent with a potential contribution of SCFA-related microbial metabolism to the observed cardiometabolic benefits.

Extensive evidence from multiple RCTs have consistently shown that tea consumption can significantly improve blood lipid metabolism. A comprehensive meta-analysis, including 10 RCTs with 411 individuals, found that black tea consumption led to a significant reduction in LDL-C levels by an average of 4.64 mg/dL, without markedly affecting TC and HDL-C levels (31). Complementing these findings, another meta-analysis of 31 trials involving 3,321 subjects reported that green tea consumption similarly resulted in a significant decrease in both TC and LDL-C levels (32). However, green tea also reduced TG levels, unlike black tea, did not reduce HDL-C levels. In addition, this analysis did not display any significant publication bias. In a randomized, double-blind study by Venkatakrishnan *et al.*, catechin-enriched oolong tea significantly reduced TC levels by 8.89%, LDL-C levels by 6.30%, and TG levels by 10.4%, while increasing HDL-C levels by 1.06-fold in mildly hypercholesterolemic subjects over 12 weeks, outperforming green tea and the placebo in lipid regulation (33). Although a plethora of animal-based research has indicated that FBT markedly ameliorates lipid metabolism, mainly through a reduction in TG and LDL-C levels, RCTs supporting these findings in humans are limited. The only previously published work by Fu *et al.*, encompassing a 120-day regimen of FBT on a small cohort of 10 individuals, demonstrated a decrease in TG, LDL-C, and TC levels, concomitantly with an elevation in HDL-C levels. Our current study partially aligns with these findings, demonstrating a robust reduction in LDL-C that was confirmed by linear mixed-effects modeling independent of confounders (34). This suggests that the lipid-lowering effects of FBT may be attributable to its unique composition, including polyphenols, polysaccharides and *Eurotium cristatum*, which might modulate lipid metabolism through various mechanisms.

Tea consumption has also been linked to improved vascular function and a reduced risk of cardiovascular diseases (5). A meta-analysis by Ras et al. (35) highlighted that tea intake boosted FMD, a key marker of vascular health, by 2.6%. Grassi et al. (36) further demonstrated that black tea significantly enhanced FMD and reduced arterial stiffness in healthy individuals. Additionally, a study by Alexopoulos et al. (37) involving 14 participants showed that green tea acutely improved endothelial function, as evidenced by increased FMD but not NMD. Our study contributed to this evidence by showing that a 12-week intervention with FBT not only improved endothelium-dependent FMD but also enhanced non-endothelium-dependent vasodilation (NMD) in healthy adults. The improvements in vascular function observed in our study may be attributed to FBT’s unique composition. These results suggest that FBT, like other tea types, offers cardioprotective benefits, possibly through mechanisms involving endothelial function modulation.

The gut microbiome has been considered a potential mediator of favorable cardiometabolic effects from tea consumption (38). Compounds from tea are not easily absorbed in the small intestine, which facilitates their interaction with gut microbiota. It is generally agreed that the small intestine only absorbs a very small percentage of tea polyphenols and polysaccharides that have been ingested, typically less than 5% (39). Many tea polyphenols and polysaccharides escape absorption in the small intestine and remain intact when they reach the colon, where they are exposed to more than 1,000 different kinds of gut bacteria. Gut microbes not only metabolize and break down tea polyphenols into smaller metabolites but also are also affected by tea polyphenols and their biological derivatives. Empirical evidence has shown that all six types of tea, including green tea, white tea, yellow tea, oolong tea, black tea, and dark tea, significantly modulated the gut microbiota of rats with a high-fat diet-induced metabolic syndrome, despite that specific detailed bacterial taxa may vary depending on the type of tea (40). Furthermore, these alterations exhibit a conspicuous correlation with the amelioration of metabolic syndrome. *In vitro* incubation of human gut microbiota has revealed that tea polyphenols and polysaccharides can directly and selectively modulate the abundance of specific bacterial species, significantly increasing *Bifidobacterium* and *Lactobacillus* while inhibiting the proliferation of *Prevotella* and *Clostridium* (41). Proof-of-principle studies from animal experiments using antibiotics and fecal transplants support the concept that gut microbiota acts as an antecedent for tea extracts to improve cardiometabolic health. In a study by Lu *et al.*, the transplantation of fecal microbiota from donor mice treated with an extract of ripened Pu-erh tea exhibited a notable impact on the recipient mice’s modulation of weight and metabolic syndrome symptoms (42). Consistently, Tang et al. (43) reported that the extract of Liupao tea prevented metabolic-associated fatty liver disease induced by a high-fat diet in ApoE^−/−^ mice through fecal microbiota transplantation. Noteworthy, Liu et al. (44) provided evidence indicating that the beneficial effects of FBT in reducing obesity and its associated metabolic disorders are counteracted by the depletion of microbiota following antibiotic treatment. Further, accumulating literature has demonstrated that the unique components found in FBT, in addition to tea polyphenols, also exert significant regulatory effects on gut microbiota. For instance, *Eurotium cristatum*, a dominant fungus in FBT production, significantly alters gut fungal and bacterial composition and increases butyrate levels fivefold in mice, thereby reducing obesity and underscoring its probiotic benefits for gut health (45, 46). The purified fraction of polysaccharides derived from FBT, specifically FBTPS-2 and FBTPS-3, also exerted anti-inflammatory and anti-obesity effects by modulating gut microbiota and enhancing SCFA production (47–50). The above evidence was mainly derived from animal experiments, while research in humans has been limited. Yuan et al.’s study on healthy individuals revealed that green tea liquid consumption can modify both oral and gut microbiota compositions (23). However, a 12-week RCT failed to observe any significant effects of green tea polyphenols on the gut microbiota of healthy participants (22). Similarly, in a randomized, placebo-controlled, double-blinded trial involving healthy postmenopausal women, fecal microbiota was not significantly influenced following a 12-month administration of a green tea polyphenol supplement (21). The inconsistency may be attributed to factors such as the study design, sample size, dosage, compliance, and depth of 16S rRNA sequencing. To the best of our knowledge, this is the first RCT to investigate the effects of FBT on gut microbiota. In this study, we observed subtle but statistically detectable shifts in the composition of the bacterial community in healthy individuals following FBT consumption, which is in line with a previous work by Huang *et al.*, highlighting the modulatory effect of post-fermented Pu-erh tea on gut microbiota in 13 healthy male volunteers (51). Our study extends the concept that FBT may affect gut microbiota and cardiometabolic health in humans.

In the current study, exploratory species-level analyses suggested an increased abundance of several bacteria with known beneficial properties, including *Blautia stercoris*, *Roseburia hominis*, *Eubacterium saphenum*, *Eubacterium* sp. AF15 50, *Alistipes dispar*, and *Lactobacillus amylolyticus*, all of which can produce SCFA from carbohydrate metabolism. Although these species-level findings did not survive FDR correction, the convergent direction of change across multiple SCFA-producing taxa and their consistent correlations with clinical outcomes (after FDR correction) lend biological plausibility to these exploratory observations. Consistently, these observations were supported by the increase in potential functional modules associated with propionate production. *Blautia*, a genus of the *Lachnospiraceae* family, plays an important role in maintaining intestine homeostasis, preventing inflammation, and promoting the metabolism of unbound lipids and glucose by producing SCFAs. The abundance of *Blautia* is negatively correlated with metabolic diseases, such as obesity, nonalcoholic fatty liver, and type 2 diabetes (52). *Roseburia hominis*, a butyrate-producing species that restores intestinal barrier function and regulates immune function, is found to be decreased in ulcerative colitis, hypertension, alcoholic fatty liver, and type 1 diabetes (53). *Eubacterium* is another SCFA-producing bacterium inversely associated with liver total TG, interleukin-2, and C-reactive protein levels. *Alistipes* is a propionate producer that plays a protective role in cardiovascular diseases, such as hypertension, atrial fibrillation, heart failure, and atherosclerosis cardiovascular disease (54). *Lactobacillus*, a famous probiotic, is known to produce bile salt hydrolase and SCFAs to inhibit cholesterol absorption (55). Short-term *Lactobacillus* supplementation has been shown to improve vascular endothelial function and reduce systemic inflammation (56). Previous animal experiments have also corroborated that administration of crude FBT extract or its bioactive constituents increases the abundance of SCFA-producing bacteria, which was accompanied by augmented SCFA levels in fecal and blood samples (48, 57–60). SCFAs play a critical role in ameliorating various risk factors associated with CMDs, including dyslipidemia, insulin resistance, inflammation, endothelial dysfunction, and vascular remodeling (61). This beneficial impact may be attributable to their ability to inhibit histone deacetylases and activate diverse free fatty acid receptors (62). We further explored potential interactions between gut microbiota and clinical outcomes. Our findings revealed significant negative correlations between these SCFA-producing bacteria and LDL-C levels, while positive correlations were observed with FMD and NMD. Taken together with previous studies, our data suggest that an increase in these SCFA-producing bacteria may partially contribute to the beneficial effect of FBT consumption on cardiometabolic health, although these species-level observations require confirmation in larger, independent cohorts.

The primary strength of our study lies in the RCT design, which established a robust framework for systematic data collection. Several limitations should be acknowledged. First, due to the distinct aroma and taste of FBT, it is not feasible to implement a blinded intervention among participants, which could potentially introduce expectation bias. A larger, double-blind, placebo-controlled randomized trial is needed to validate our findings. Second, while we employed metagenomic sequencing to detect species-level differences, our study did not include fecal or serum SCFA quantification, as the sample collection protocol was not optimized for metabolomic analysis of volatile short-chain fatty acids. Consequently, while our metagenomic data and functional pathway analysis support an enrichment of SCFA-producing bacteria and related biosynthetic pathways, we cannot directly confirm increased SCFA production or establish a causal link between SCFA-producing bacteria and the observed cardiometabolic improvements. Future studies incorporating targeted SCFA metabolomics with appropriate sample preservation protocols are warranted to validate this proposed hypothesis. Third, this study does not allow us to conclude the direct causality between gut microbiota alterations and the observed health benefits, which should be further verified by germ-free animals and fecal transplant experiments. Fourth, the 12-week intervention may be insufficient for the gut microbiota to reach a new steady state, and the lack of post-withdrawal follow-up limits our understanding of the persistence of microbiota changes after FBT cessation. Fifth, the identification of differentially abundant bacterial species and GMM pathway was based on permuspliner *p*-values without cross-species multiple testing correction. Although SplinectomeR’s permutation-based framework provides statistical control for longitudinal comparisons, neither the species-level nor the pathway-level findings survived Benjamini–Hochberg FDR correction, and these results should therefore be interpreted as exploratory and require validation in independent cohorts with adequate sample sizes. Finally, the composition of FBT is complex, and our study cannot decipher the involved bioactive compounds responsible for modulating gut microbiota. More work is needed to elucidate the biological activity of FBT and identify the bioactive compounds and their corresponding metabolites.

## Conclusion

In conclusion, our study demonstrated a beneficial effect of FBT consumption on the lipid profile and vascular function. Exploratory species-level analyses suggested that FBT consumption is associated with favorable alterations in gut microbiota, particularly by augmenting the abundance of SCFA-producing bacteria, although these findings require validation in larger studies. This suggests a potential functional role for these microbial changes in the beneficial effects of FBT consumption in humans. These findings align with the increasing recognition of tea consumption as a low-risk, cost-effective preventive strategy against CMDs. Nevertheless, future well-designed trials with a larger sample size; a randomized, double-blind, placebo-controlled, multicenter design, alongside comprehensive multiomics analyses are needed to establish a clearer mechanistic understanding of the potential crosstalk between FBT consumption and gut microbiota, and their impacts on host cardiometabolic health.

## Data Availability

The metagenomic sequencing data presented in this study have been deposited in the NCBI BioProject Database under accession code PRJNA1083321. (https://www.ncbi.nlm.nih.gov/bioproject/PRJNA1083321).

## Footnotes

1
https://www.r-project.org

## Supplementary material

SUPPLEMENTARY FIGURE 1 Impact of Fuzhuan brick tea on the gut microbiome composition at the phylum levels in healthy adults over time.

SUPPLEMENTARY FIGURE 2 Longitudinal differences in the bacteria between the two groups over time using the SplinectomeR permuspliner function. These species were identified using permuspliner with unadjusted *p* < 0.05. After Benjamini-Hochberg FDR correction across all 77 species, none remained statistically significant (see Supplementary Table 5). All species-level findings should be interpreted as exploratory.

SUPPLEMENTARY FIGURE 3 Three-pronged association heatmap of bacterial species, GMM pathways, and clinical outcomes at week 12 alone. The left panel denotes the family-level and phylum-level taxonomy and directionality of enrichment in the FBT group (red) and control group (green) for species. The centered heatmap denotes the Spearman correlation between each species and each GMM pathway (red: positive correlation; blue: negative correlation). The right panel denotes the Spearman correlations between bacterial species and clinical parameters, colored by positive (pink), negative (green), or nonsignificant correlation (white). The bottom panel denotes the significant association between the GMM pathways and clinical parameters (cyan: positive correlation; brown: negative correlation). * denotes FDR *p* < 0.05; ** denotes FDR *p* < 0.01.

## References

[1] Virani SS Alonso A Benjamin EJ Bittencourt MS Callaway CW Carson AP . Heart disease and stroke statistics—2020 update: a report from the American Heart Association. Circulation. (2020) 141:e139–596. doi: 10.1161/CIR.0000000000000757, 31992061

[2] Sattar N Gill JMR Alazawi W. Improving prevention strategies for cardiometabolic disease. Nat Med. (2020) 26:320–5. doi: 10.1038/s41591-020-0786-7, 32152584

[3] Wang W Liu Y Li Y Luo B Lin Z Chen K . Dietary patterns and cardiometabolic health: clinical evidence and mechanism. Medcomm. (2023) 4:e212. doi: 10.1002/mco2.212, 36776765 PMC9899878

[4] Pan SY Nie Q Tai HC Song XL Tong YF Zhang LJF . Tea and tea drinking: China’s outstanding contributions to the mankind. Chin Med. (2022) 17:27. doi: 10.1186/s13020-022-00571-1, 35193642 PMC8861626

[5] Wang X Liu F Li J Yang X Chen J Cao J . Tea consumption and the risk of atherosclerotic cardiovascular disease and all-cause mortality: the China-PAR project. Eur J Prev Cardiol. (2020) 27:1956–63. doi: 10.1177/2047487319894685, 31914807

[6] Xiang Y Xu H Chen H Tang D Huang Z Zhang Y . Tea consumption and attenuation of biological aging: a longitudinal analysis from two cohort studies. Lancet Reg Health West Pac. (2024) 42:100955. doi: 10.1016/j.lanwpc.2023.100955, 38075587 PMC10700389

[7] Brody H. Tea. Nature. (2019) 566:S1–1. doi: 10.1038/d41586-019-00394-5, 30728511

[8] Zhang L Zhang Z Zhou Y Ling T Wan X. Chinese dark teas: postfermentation, chemistry and biological activities. Food Res Int. (2013) 53:600–7. doi: 10.1016/j.foodres.2013.01.016

[9] Xu A Wang Y Wen J Liu P Liu Z Li Z. Fungal community associated with fermentation and storage of Fuzhuan brick-tea. Int J Food Microbiol. (2011) 146:14–22. doi: 10.1016/j.ijfoodmicro.2011.01.024, 21345511

[10] Chen G Peng Y Xie M Xu W Chen C Zeng X . A critical review of Fuzhuan brick tea: processing, chemical constituents, health benefits and potential risk. Crit Rev Food Sci Nutr. (2023) 63:5447–64. doi: 10.1080/10408398.2021.2020718, 34964426

[11] Chen Y Chen J Chen R Xiao L Wu X Hu L . Comparison of the fungal community, chemical composition, antioxidant activity, and taste characteristics of Fu brick tea in different regions of China. Front Nutr. (2022) 9:900138. doi: 10.3389/fnut.2022.900138, 35656159 PMC9152283

[12] Du Y Yang W Yang C Yang X. A comprehensive review on microbiome, aromas and flavors, chemical composition, nutrition and future prospects of Fuzhuan brick tea. Trends Food Sci Technol. (2022) 119:452–66. doi: 10.1016/j.tifs.2021.12.024

[13] Bai Y Zeng Z Xie Z Chen G Chen D Sun Y . Effects of polysaccharides from Fuzhuan brick tea on immune function and gut microbiota of cyclophosphamide-treated mice. J Nutr Biochem. (2022) 101:108947. doi: 10.1016/j.jnutbio.2022.108947, 35017006

[14] Wang Y Xu A Liu P Li Z. Effects of Fuzhuan brick-tea water extract on mice infected with *E. coli* O157:H7. Nutrients. (2015) 7:5309–26. doi: 10.3390/nu7075218, 26140539 PMC4516995

[15] Gabriel CL Ferguson JF. Gut microbiota and microbial metabolism in early risk of cardiometabolic disease. Circ Res. (2023) 132:1674–91. doi: 10.1161/CIRCRESAHA.123.322055, 37289901 PMC10254080

[16] Chakaroun RM Olsson LM Bäckhed F. The potential of tailoring the gut microbiome to prevent and treat cardiometabolic disease. Nat Rev Cardiol. (2023) 20:217–35. doi: 10.1038/s41569-022-00771-0, 36241728

[17] Morelli MB Wang X Santulli G. Functional role of gut microbiota and PCSK9 in the pathogenesis of diabetes mellitus and cardiovascular disease. Atherosclerosis. (2019) 289:176–8. doi: 10.1016/j.atherosclerosis.2019.07.023, 31431286 PMC6790279

[18] Wan Y Tang J Li J Li J Yuan J Wang F . Contribution of diet to gut microbiota and related host cardiometabolic health: diet-gut interaction in human health. Gut Microbes. (2020) 11:603–9. doi: 10.1080/19490976.2019.1697149, 31964203 PMC7524383

[19] Liu YC Li XY Shen L. Modulation effect of tea consumption on gut microbiota. Appl Microbiol Biotechnol. (2020) 104:981–7. doi: 10.1007/s00253-019-10306-2 31853562

[20] Bond T Derbyshire E. Tea compounds and the gut microbiome: findings from trials and mechanistic studies. Nutrients. (2019) 11:2364. doi: 10.3390/nu11102364, 31623411 PMC6835862

[21] Janssens PLHR Penders J Hursel R Budding AE Savelkoul PHM Westerterp-Plantenga MS. Long-term green tea supplementation does not change the human gut microbiota. PLoS One. (2016) 11:e0153134. doi: 10.1371/journal.pone.0153134, 27054321 PMC4824458

[22] Zhou Y Zhang N Arikawa AY Chen C. Inhibitory effects of green tea polyphenols on microbial metabolism of aromatic amino acids in humans revealed by metabolomic analysis. Meta. (2019) 9:96. doi: 10.3390/metabo9050096, 31083533 PMC6571926

[23] Yuan X Long Y Ji Z Gao J Fu T Yan M . Green tea liquid consumption alters the human intestinal and oral microbiome. Mol Nutr Food Res. (2018) 62:e1800178. doi: 10.1002/mnfr.201800178, 29750437 PMC6033105

[24] Wensel CR Pluznick JL Salzberg SL Sears CL. Next-generation sequencing: insights to advance clinical investigations of the microbiome. J Clin Invest. (2022) 132:e154944. doi: 10.1172/JCI154944, 35362479 PMC8970668

[25] Gambardella J Castellanos V Santulli G. Standardizing translational microbiome studies and metagenomic analyses. Cardiovasc Res. (2021) 117:640–2. doi: 10.1093/cvr/cvaa175, 32569375 PMC7898946

[26] Liang Y Wu F Wu D Zhu X Gao X Hu X . Fu loose tea administration ameliorates obesity in high-fat diet-fed C57BL/6J mice: a comparison with Fu brick tea and orlistat. Foods. (2024) 13:206. doi: 10.3390/foods13020206, 38254507 PMC10815023

[27] Blanco-Míguez A Beghini F Cumbo F McIver LJ Thompson KN Zolfo M . Extending and improving metagenomic taxonomic profiling with uncharacterized species using MetaPhlAn 4. Nat Biotechnol. (2023) 41:1633–44. doi: 10.1038/s41587-023-01688-w, 36823356 PMC10635831

[28] Shields-Cutler RR Al-Ghalith GA Yassour M Knights D. SplinectomeR enables group comparisons in longitudinal microbiome studies. Front Microbiol. (2018) 9:785. doi: 10.3389/fmicb.2018.00785, 29740416 PMC5924793

[29] Vieira-Silva S Falony G Darzi Y Lima-Mendez G Garcia Yunta R Okuda S . Species–function relationships shape ecological properties of the human gut microbiome. Nat Microbiol. (2016) 1:16088. doi: 10.1038/nmicrobiol.2016.88, 27573110

[30] Langmead B Salzberg SL. Fast gapped-read alignment with bowtie 2. Nat Methods. (2012) 9:357–9. doi: 10.1038/nmeth.1923, 22388286 PMC3322381

[31] Zhao Y Asimi S Wu K Zheng J Li D. Black tea consumption and serum cholesterol concentration: systematic review and meta-analysis of randomized controlled trials. Clin Nutr. (2015) 34:612–9. doi: 10.1016/j.clnu.2014.06.003, 24972454

[32] Xu R Yang K Li S Dai M Chen G. Effect of green tea consumption on blood lipids: a systematic review and meta-analysis of randomized controlled trials. Nutr J. (2020) 19:48. doi: 10.1186/s12937-020-00557-5, 32434539 PMC7240975

[33] Venkatakrishnan K Chiu H-F Cheng J-C Chang Y-H Lu Y-Y Han Y-C . Comparative studies on the hypolipidemic, antioxidant and hepatoprotective activities of catechin-enriched green and oolong tea in a double-blind clinical trial. Food Funct. (2018) 9:1205–13. doi: 10.1039/C7FO01449J, 29384173

[34] Fu D Ryan EP Huang J Liu Z Weir TL Snook RL . Fermented *Camellia sinensis*, Fu Zhuan tea, regulates hyperlipidemia and transcription factors involved in lipid catabolism. Food Res Int. (2011) 44:2999–3005. doi: 10.1016/j.foodres.2011.07.008

[35] Ras RT Zock PL Draijer R. Tea consumption enhances endothelial-dependent vasodilation; a meta-analysis. PLoS One. (2011) 6:e16974. doi: 10.1371/journal.pone.0016974, 21394199 PMC3048861

[36] Grassi D Mulder TP Draijer R Desideri G Molhuizen HO Ferri C. Black tea consumption dose-dependently improves flow-mediated dilation in healthy males. J Hypertens. (2009) 27:774–81. doi: 10.1097/HJH.0b013e328326066c, 19516176

[37] Alexopoulos N Vlachopoulos C Aznaouridis K Baou K Vasiliadou C Pietri P . The acute effect of green tea consumption on endothelial function in healthy individuals. Eur J Cardiovasc Prev Rehabil. (2008) 15:300–5. doi: 10.1097/HJR.0b013e3282f4832f, 18525384

[38] Li Q Van De Wiele T. Gut microbiota as a driver of the interindividual variability of cardiometabolic effects from tea polyphenols. Crit Rev Food Sci Nutr. (2023) 63:1500–26. doi: 10.1080/10408398.2021.1965536, 34515591

[39] Manach C Scalbert A Morand C Rémésy C Jiménez L. Polyphenols: food sources and bioavailability. Am J Clin Nutr. (2004) 79:727–47. doi: 10.1093/ajcn/79.5.727, 15113710

[40] Zhou F Zhu M Tang J Ou-yang J Shang B Liu C . Six types of tea extracts attenuated high-fat diet-induced metabolic syndrome via modulating gut microbiota in rats. Food Res Int. (2022) 161:111788. doi: 10.1016/j.foodres.2022.111788, 36192879

[41] Sun H Chen Y Cheng M Zhang X Zheng X Zhang Z. The modulatory effect of polyphenols from green tea, oolong tea and black tea on human intestinal microbiota in vitro. J Food Sci Technol. (2018) 55:399–407. doi: 10.1007/s13197-017-2951-7, 29358833 PMC5756227

[42] Lu X Liu J Zhang N Fu Y Zhang Z Li Y . Ripened Pu-erh tea extract protects mice from obesity by modulating gut microbiota composition. J Agric Food Chem. (2019) 67:6978–94. doi: 10.1021/acs.jafc.8b04909, 31070363

[43] Tang W Yuan M Li Z Lin Q Zhen Y Li Z . Polyphenol-rich Liupao tea extract prevents high-fat diet-induced MAFLD by modulating the gut microbiota. Nutrients. (2022) 14:4930. doi: 10.3390/nu14224930, 36432617 PMC9697786

[44] Liu D Huang J Luo Y Wen B Wu W Zeng H . Fuzhuan brick tea attenuates high-fat diet-induced obesity and associated metabolic disorders by shaping gut microbiota. J Agric Food Chem. (2019) 67:13589–604. doi: 10.1021/acs.jafc.9b05833, 31735025

[45] Kang D Su M Duan Y Huang Y. Eurotium cristatum, a potential probiotic fungus from Fuzhuan brick tea, alleviated obesity in mice by modulating gut microbiota. Food Funct. (2019) 10:5032–45. doi: 10.1039/c9fo00604d, 31359019

[46] Wang X Liu J Wei J Zhang Y Xu Y Yue T . Protective mechanism of Eurotium amstelodami from Fuzhuan brick tea against colitis and gut-derived liver injury induced by dextran sulfate sodium in C57BL/6 mice. Nutrients. (2024) 16:1178. doi: 10.3390/nu16081178, 38674869 PMC11054642

[47] Chen G Zeng Z Xie M Peng Y Zhou W Xu W . Fermentation characteristics and probiotic activity of a purified fraction of polysaccharides from Fuzhuan brick tea. Food Sci Human Wellness. (2022) 11:727–37. doi: 10.1016/j.fshw.2021.12.030

[48] Zhu M Ouyang J Zhou F Zhao C Zhu W Liu C . Polysaccharides from Fu brick tea ameliorate obesity by modulating gut microbiota and gut microbiota-related short chain fatty acid and amino acid metabolism. J Nutr Biochem. (2023) 118:109356. doi: 10.1016/j.jnutbio.2023.109356, 37087075

[49] Chen G Wang M Zeng Z Xie M Xu W Peng Y . Fuzhuan brick tea polysaccharides serve as a promising candidate for remodeling the gut microbiota from colitis subjects in vitro: fermentation characteristic and anti-inflammatory activity. Food Chem. (2022) 391:133203. doi: 10.1016/j.foodchem.2022.133203, 35597036

[50] Chen G Xie M Wan P Chen D Dai Z Ye H . Fuzhuan brick tea polysaccharides attenuate metabolic syndrome in high-fat diet induced mice in association with modulation in the gut microbiota. J Agric Food Chem. (2018) 66:2783–95. doi: 10.1021/acs.jafc.8b00296, 29514453

[51] Huang F Zheng X Ma X Jiang R Zhou W Zhou S . Theabrownin from Pu-erh tea attenuates hypercholesterolemia via modulation of gut microbiota and bile acid metabolism. Nat Commun. (2019) 10:4971. doi: 10.1038/s41467-019-12896-x, 31672964 PMC6823360

[52] Liu X Mao B Gu J Wu J Cui S Wang G . Blautia—a new functional genus with potential probiotic properties? Gut Microbes. (2021) 13:1–21. doi: 10.1080/19490976.2021.1875796, 33525961 PMC7872077

[53] Tamanai-Shacoori Z Smida I Bousarghin L Loreal O Meuric V Fong SB . *Roseburia* spp.: a marker of health? Future Microbiol. (2017) 12:157–70. doi: 10.2217/fmb-2016-0130, 28139139

[54] Parker BJ Wearsch PA Veloo ACM Rodriguez-Palacios A. The genus Alistipes: gut bacteria with emerging implications to inflammation, cancer, and mental health. Front Immunol. (2020) 11:00906. doi: 10.3389/fimmu.2020.00906, 32582143 PMC7296073

[55] Minj J Chandra P Paul C Sharma RK. Bio-functional properties of probiotic Lactobacillus: current applications and research perspectives. Crit Rev Food Sci Nutr. (2021) 61:2207–24. doi: 10.1080/10408398.2020.1774496, 32519883

[56] Malik M Suboc TM Tyagi S Salzman N Wang J Ying R . *Lactobacillus plantarum* 299v supplementation improves vascular endothelial function and reduces inflammatory biomarkers in men with stable coronary artery disease. Circ Res. (2018) 123:1091–102. doi: 10.1161/CIRCRESAHA.118.313565, 30355158 PMC6205737

[57] Qi B Ren D Li T Niu P Zhang X Yang X . Fu brick tea manages HFD/STZ-induced type 2 diabetes by regulating the gut microbiota and activating the IRS1/PI3K/Akt signaling pathway. J Agric Food Chem. (2022) 70:8274–87. doi: 10.1021/acs.jafc.2c02400, 35767631

[58] Du H Shi L Yan T Wang Q Wang Y Zhao Y . Fu brick tea protects against high-fat diet-induced obesity phenotypes via promoting adipose browning and thermogenesis in association with gut microbiota. Food Funct. (2022) 13:11111–24. doi: 10.1039/d2fo02063g, 36200599

[59] Ou-yang J Li X Liu C Ou-yang J Tang J Liu Q . Moringa-Fu brick tea extract attenuated high-fat diet-induced obesity via modulating bile acid metabolism and gut microbiota in rats. J Funct Foods. (2023) 109:105766. doi: 10.1016/j.jff.2023.105766

[60] Wu D Zhao H Guo L Liu X Liang Y Liu Q . Fu brick tea as a staple food supplement attenuates high fat diet induced obesity in mice. Foods. (2023) 12:4488. doi: 10.3390/foods12244488, 38137292 PMC10743230

[61] Nogal A Valdes AM Menni C. The role of short-chain fatty acids in the interplay between gut microbiota and diet in cardio-metabolic health. Gut Microbes. (2021) 13:1897212. doi: 10.1080/19490976.2021.1897212, 33764858 PMC8007165

[62] Feng Y Xu D. Short-chain fatty acids are potential goalkeepers of atherosclerosis. Front Pharmacol. (2023) 14:1271001. doi: 10.3389/fphar.2023.1271001, 38027009 PMC10679725

