# Supplementary material for: Daily consumption of Fuzhuan brick tea improves lipid profile and vascular function associated with modulation in gut microbiota composition in healthy adults: a pilot randomized controlled trial
Source: Front Nutr. 2026 Sep 28;13:1827773. doi: 10.3389/fnut.2026.1827773 (PMC13656248; doi:10.3389/fnut.2026.1827773)
Supplement: Supplementary file 1 [file Table_1.docx]

**Supplementary Table 1. Baseline characteristics between completers and non-completers**

| **Variable** | **Completers (n=110)** | **Non-completers (n=10)** | **Statistic Value** | **P value** |
| --- | --- | --- | --- | --- |
| Age (years), Mean ± SD | 53.21±7.21 | 54.53±8.99 | 0.545 | 0.587 ^a^ |
| Sex, n (male/female) | 55/55 | 6/4 | 0.367 | 0.743 ^b^ |
| Alcohol use, n (y/n) | 45/65 | 3/7 | 0.455 | 0.738 ^b^ |
| Smokers, n (y/n) | 34/76 | 2/8 | 0.519 | 0.721 ^b^ |
| BMI, kg/m² | 25.24±3.02 | 25.20±3.14 | 0.040 | 0.968 ^a^ |
| SBP, mmHg | 124.26±12.43 | 123.78±8.83 | 0.120 | 0.905 ^a^ |
| DBP, mmHg | 78.84±7.51 | 81.55±10.65 | 1.053 | 0.294 ^a^ |
| HbA1c, % | 5.77±0.29 | 5.79±0.17 | 0.238 | 0.812 ^a^ |
| BUN, mmol/L | 5.37±1.48 | 5.41±0.54 | 0.076 | 0.940 ^a^ |
| SCr, μmol/L | 58.72±11.57 | 55.74±10.43 | 0.788 | 0.433 ^a^ |
| AST, U/L | 22.30±6.90 | 23.25±4.82 | 0.425 | 0.672 ^a^ |
| ALT, U/L | 20.14±10.82 | 18.59±9.63 | 0.437 | 0.663 ^a^ |
| Total cholesterol, mmol/L | 5.07±1.03 | 4.95±0.80 | 0.346 | 0.730 ^a^ |
| Triglycerides, mmol/L | 1.85±1.29 | 1.76±1.00 | 0.214 | 0.831 ^a^ |
| LDL cholesterol, mmol/L | 3.12±0.83 | 3.46±0.48 | 1.268 | 0.207 ^a^ |
| HDL cholesterol, mmol/L | 1.17±0.31 | 1.14±0.21 | 0.390 | 0.697 ^a^ |
| Physical activity, MET-min/week | 2067.72±559.75 | 2153.91±476.41 | 0.471 | 0.638 ^a^ |

*a: Independent-samples t-test; b: Chi-square test or Fisher's exact test.*
