# Supplementary material for: Daily consumption of Fuzhuan brick tea improves lipid profile and vascular function associated with modulation in gut microbiota composition in healthy adults: a pilot randomized controlled trial
Source: Front Nutr. 2026 Sep 28;13:1827773. doi: 10.3389/fnut.2026.1827773 (PMC13656248; doi:10.3389/fnut.2026.1827773)
Supplement: Supplementary file 2 [file Table_4.docx]

**Supplementary Table 4. ANCOVA sensitivity analysis adjusting for baseline values**

| **Outcome** | **Analysis** | **Adjusted mean difference** | **95% CI** | **P value** | **Cohen's d** |
| --- | --- | --- | --- | --- | --- |
| **FMD** | Model 1 | 6.27 | 3.40, 9.14 | **<0.001** | 0.59 |
|  | Model 2 | 6.45 | 3.44, 9.45 | **<0.001** | — |
| **NMD** | Model 1 | 5.92 | 2.63, 9.20 | **<0.001** | 0.49 |
|  | Model 2 | 5.95 | 2.53, 9.38 | **<0.001** | — |
| **LDL-C (mmol/L)** | Model 1 | −0.43 | −0.61, −0.26 | **<0.001** | −0.54 |
|  | Model 2 | −0.48 | −0.66, −0.30 | **<0.001** | — |
| **TC (mmol/L)** | Model 1 | 0.04 | −0.17, 0.25 | 0.692 | 0.05 |
|  | Model 2 | −0.002 | −0.22, 0.21 | 0.984 | — |
| **TG (mmol/L)** | Model 1 | −0.07 | −0.42, 0.27 | 0.672 | −0.08 |
|  | Model 2 | −0.09 | −0.45, 0.27 | 0.624 | — |
| **HDL-C (mmol/L)** | Model 1 | 0.05 | −0.01, 0.11 | 0.119 | 0.16 |
|  | Model 2 | 0.04 | −0.02, 0.11 | 0.199 | — |

ANCOVA, analysis of covariance; FMD, flow-mediated dilation; NMD, nitroglycerin-mediated dilation; LDL-C, low-density lipoprotein cholesterol; TC, total cholesterol; TG, triglyceride; HDL-C, high-density lipoprotein cholesterol. Model 1: ANCOVA adjusted for baseline value of the corresponding outcome only. Model 2: ANCOVA further adjusted for age, sex, and BMI. Cohen's d is reported for Model 1 only; Model 2 did not include Cohen's d calculation.
